# Long-term clinical course of adult-onset refractory epilepsy in cardiofaciocutaneous syndrome with a pathogenic *MAP2K1* variant: a case report

**DOI:** 10.3389/fgene.2024.1410979

**Published:** 2024-07-17

**Authors:** Rie Tsuburaya-Suzuki, Sachiko Ohori, Kohei Hamanaka, Atsushi Fujita, Naomichi Matsumoto, Masako Kinoshita

**Affiliations:** ^1^ Department of Pediatric Neurology, National Hospital Organization Utano National Hospital, Kyoto, Japan; ^2^ Department of Pediatrics, St. Joseph Medical and Welfare Center for Children, Kyoto, Japan; ^3^ Department of Human Genetics, Yokohama City University Graduate School of Medicine, Yokohama, Japan; ^4^ Department of Neurology, National Hospital Organization Utano National Hospital, Kyoto, Japan

**Keywords:** cardiofaciocutaneous syndrome, CFC syndrome, *MAP2K1*, epilepsy, neurodevelopmental disorders, drug-induced hypersensitivity syndrome

## Abstract

Cardiofaciocutaneous syndrome (CFC) is a rare genetic disorder that presents with cardiac, craniofacial, and cutaneous symptoms, and is often accompanied by neurological abnormalities, including neurodevelopmental disorders and epilepsy. Regarding epilepsy in CFC, the onset of seizures commonly occurs in childhood. Since research data has mainly been collected from young patients with relatively short observation period, there is insufficient information regarding adult-onset epilepsy in CFC. Here, we report the long-term clinical course of epilepsy and other complications in a 45-year-old female with genetically confirmed CFC carrying a pathogenic *de novo* heterozygous variant of *MAP2K1*, c.389 A>G (p.Tyr130Cys). The patient presented psychomotor delay from infancy and had severe intellectual disability with autistic features. At the age of 30, she first developed combined generalized and focal epilepsy that was resistant to anti-seizure medication. Her refractory epilepsy was fairly controlled with a combination of three anti-seizure medications, especially lacosamide, which effectively suppressed both generalized and focal seizures. The present case provides detailed information regarding the clinical course and treatment of adult-onset epilepsy, which may be useful for optimal treatment and prognostic prediction of CFC.

## 1 Introduction

Cardiofaciocutaneous syndrome (CFC, OMIM#115150, ORPHA:1340) is characterized by congenital cardiac defects, craniofacial features, and ectodermal abnormalities (Pierpont et al., 2014). CFC belongs to a group of syndromes termed RASopathies, which occur due to variants of genes involved in the RAS/mitogen-activated protein kinase (RAS/MAPK) pathway, including *BRAF*, *MAP2K1*, *MAP2K2*, and *KRAS* (Niihori et al., 2006; Rodriguez-Viciana et al., 2006; Rauen, 2013). CFC is often accompanied by intellectual disabilities and neurological complications, including epilepsy (Pierpont et al., 2014). A previous study on CFC reported that 55% of the patients developed epilepsy, and the age at which seizure onset occurred ranged between 1 month and 21 years, with a mean age of 5.1 years (Pierpont et al., 2022). Case reports have provided detailed description of infantile epileptic spasms in CFC (Aizaki et al., 2011; Adachi et al., 2012); however, little information is available regarding adult-onset epilepsy.

In the most common genotype with *BRAF* variants, a genotype-phenotype correlation has been reported between the structure and function of variants and the severity of epilepsy (Battaglia et al., 2021). Due to the rarity of *MAP2K1* related-CFC, limited information is available regarding the epileptic features of *MAP2K1* variants.

Herein, we present the case of an adult patient with CFC caused by pathogenic *MAP2K1* variant, who manifested as adult-onset refractory epilepsy. This report provides detailed information regarding epilepsy and other complications of CFC over a long period of time, which may aid in better understanding of therapeutic strategies and prognosis.

## 2 Case description

The patient was a female of Japanese origin born at term as the first child of a healthy non-consanguineous couple following an uneventful pregnancy. In the prenatal period, polyhydramnios wasn’t detected. No abnormalities were recognized in the neonatal period. During early infancy, the patient experienced feeding difficulties with gastroesophageal reflux. Her mother described her as a stiff baby. At 32 months of age, she was diagnosed with idiopathic intellectual disability, and physical therapy was initiated as she had not achieved milestones like rolling-over or crawling by that age. At 4 years of age, she finally acquired the ability to walk independently and speak few meaningful words but needed total assistance for her daily activities and attended a special needs school. No underlying diseases were found despite neurological consultations.

At 30 years of age, the patient developed generalized convulsions and visited our adult epilepsy clinic. At 45 years of age, genetic analyses revealed a pathogenic *de novo* heterozygous *MAP2K1* variant. Thus, we analyzed the long-term clinical course of epilepsy and other complications. This study was conducted according to the principles of the Declaration of Helsinki. The Institutional Ethics Review Board approved the study design. Written informed consent was obtained from the patient for publication of the case report and accompanying images.

## 3 Diagnostic assessment

**FIGURE 1:**
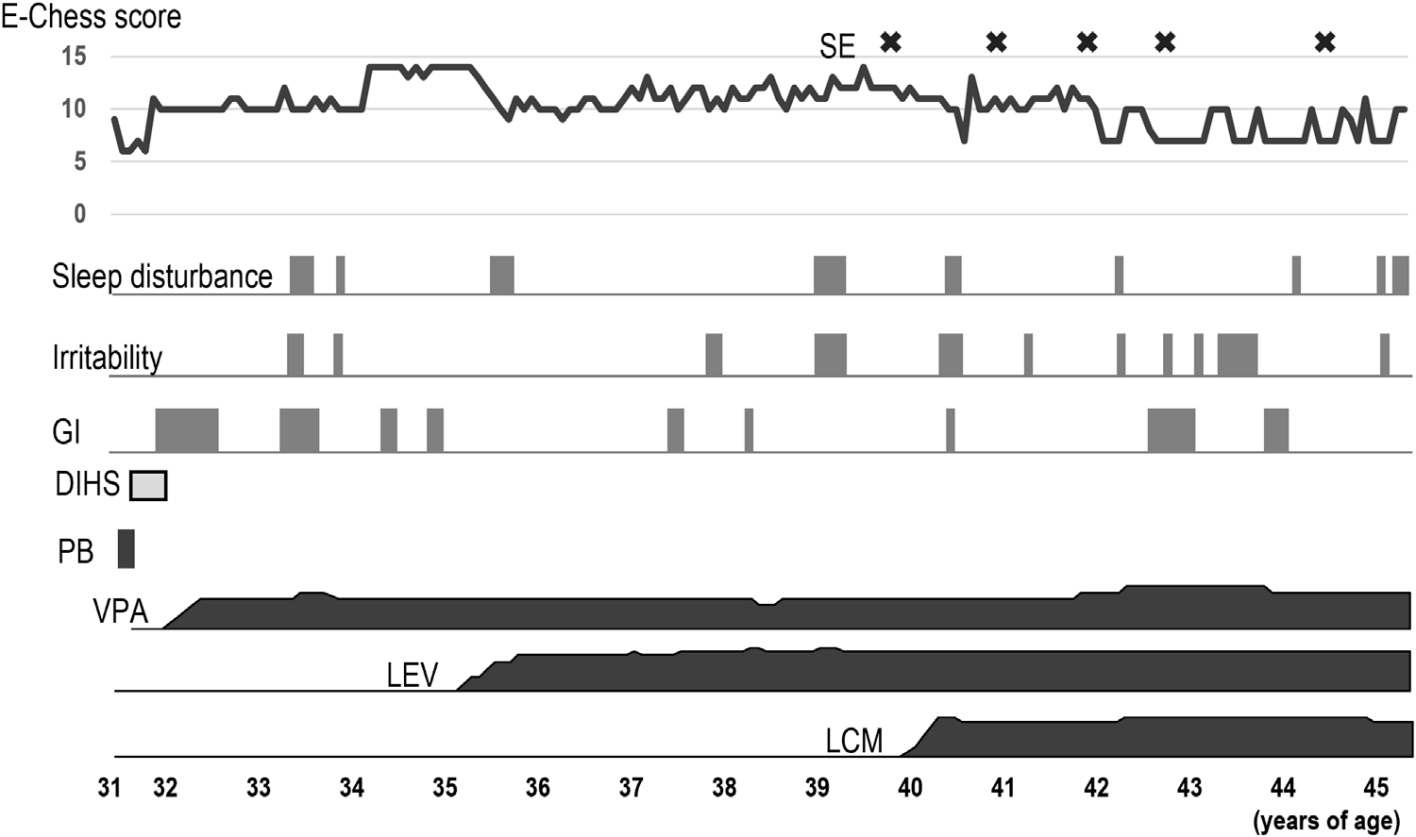
Clinical course of the patient. Severity of epilepsy was evaluated using the E-Chess scoring system, which comprises five variables; 1) frequency of seizures, 2) time period over which seizures occurred, 3) number of seizure types, 4) number of anticonvulsants used, and 5) response to treatment. A higher total score indicates greater severity. The scores were derived from the medical records. After adding LCM, E-Chess scores lowered. However, status epilepticus occasionally occurred after the age of 40 years. Sleep disturbance and irritability worsened intermittently. The patient sometimes had gastrointestinal symptoms including nausea and vomiting, and developed ileus. DIHS: drug-induced hypersensitivity syndrome, E-Chess: Early Childhood Epilepsy Severity Scale, GI: gastrointestinal symptoms, LCM: lacosamide, LEV: levetiracetam, PB: phenobarbital, SE: Status epilepticus, VPA: valproic acid.

Figure 1 shows the clinical course of epilepsy according to the Early Childhood Epilepsy Severity Scale (E-Chess) scoring system (Humphrey et al., 2008; Pierpont et al., 2022).

At 30 years of age, the patient developed recurrent generalized tonic-clonic convulsions. She had a history of simple febrile convulsions at 1 year of age, but no other risk factors for epilepsy were identified. She exhibited three types of seizures: generalized tonic-clonic, focal to bilateral tonic-clonic, and focal impaired awareness seizures. An interictal electroencephalogram revealed background slowing along with generalized and multifocal epileptiform discharges. Based on these findings, she was diagnosed with combined generalized and focal epilepsy. Twenty days after starting a small dose of phenobarbital (PB, 60 mg), she developed systemic rash, high fever, lymphadenopathy, and mucosal lesions involving the conjunctiva and oral mucosa. The cutaneous lesions developed into erythematous macules that progressed to flaccid blisters. Laboratory investigations revealed eosinophilia, acute liver dysfunction, and elevated C-reactive protein levels. The drug concentration of PB was within the therapeutic range. After cessation of PB, drug-induced hypersensitivity syndrome (DIHS) was treated with high-dose methylprednisolone pulse therapy. Since frequent seizures affected her daily life, anti-seizure medication was carefully resumed. Valproic acid (VPA, 100–700 mg) was partially effective in reducing the frequency of seizures but caused appetite loss. Although adding levetiracetam (LEV, 250–1500 mg) controlled the focal seizures, worsening sleep disturbances and irritability hindered the increase in doses. Lacosamide (LCM, 50–400 mg) was effective in controlling all types of seizures without any side effects. Finally, her seizures decreased to generalized convulsions every few months and several focal impaired awareness seizures per month with combination therapy of VPA, LEV, and LCM. She gradually regressed after her early 30 s and required more support for activities of daily living.

As for other comorbidities, the patient had bilateral juvenile cataracts and glaucoma. She underwent surgery for glaucoma at the age of 34 years and an ophthalmectomy of the left eye due to corneal perforation at 44 years of age. Compression fractures occurred in the 12th thoracic and 2nd lumbar vertebrae at 40 years of age, and bisphosphonates were initiated for osteoporosis. Ileus occurred at 42 years of age, and she required regular bowel control with enema. At 22 years of age when intravenous sedation was performed with pentazocine and hydroxyzine hydrochloride during examination for melena, she suddenly exhibited dyspnea, tachycardia, rapid elevation in temperature, and muscle rigidity. She was tentatively diagnosed and treated as neuroleptic malignant syndrome (NMS). Later, the same adverse reaction relapsed after taking brotizolam orally. After those events, she had not taken any medication until her first visit to our hospital.

**FIGURE 2:**
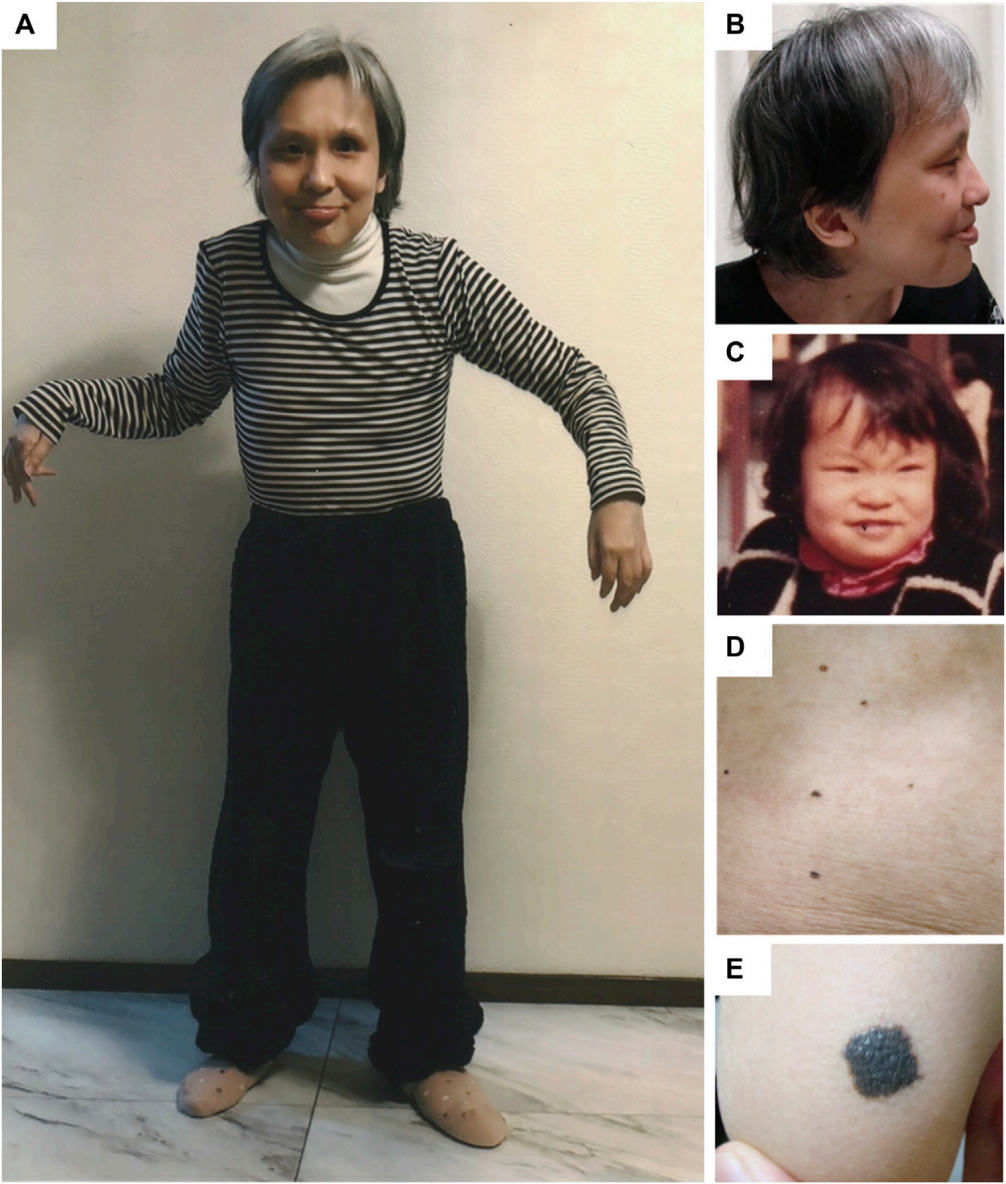
Clinical features of the patient. At 45 years of age, the patient looked older than her actual age. Frontal **(A)** and lateral **(B)** views showing prominent forehead and bitemporal narrowing, sparse eyebrows and eyelashes, hypertelorism and epicanthal folds, ptosis in the right eye and ocular prosthesis in the left eye, along with low-set and posterior-rotated ears. Joint contractures were evident in elbows, hip joints, and knees. The high anterior hairline, sparse eyebrows, ptosis, and short nose with depressed bridge are apparent from childhood **(C)**. Multiple melanocytic naevi were found on her abdomen **(D)** and right elbow **(E)**.

At 45 years of age, the height and weight of the patient were 140 cm (−3.5 SD) and 34 kg (−2.3 SD), respectively. Head circumference was 54 cm (−0.1 SD), which was relatively large. She exhibited characteristic facial features, musculoskeletal symptoms such as kyphosis and joint contractures, and cutaneous symptoms as melanocytic nevi (Figure 2). She could only ambulate with assistance and used a wheelchair for long distances. She could eat orally but needed continuous encouragement and support to have adequate food and required maximal assistance for other self-care. She had severe intellectual disability with autistic stereotyped repetitive behaviors and could communicate with gestures and several simple words. Electrocardiography revealed no abnormal findings such as arrhythmias. Echocardiography revealed mild regurgitation of the mitral and tricuspid valves with normal wall motion and no ventricular dilatation.

Genetic analyses were performed after obtaining written informed consent from her parents. Chromosomal analysis revealed a normal karyotype. Whole exome sequencing of the patient and her parents using methods described previously (Hamanaka et al., 2022) revealed a pathogenic *de novo* heterozygous *MAP2K1* variant, c.389 A>G (p.Tyr130Cys) (MIM#615279, NM_002755.4), which was previously reported (Rodriguez-Viciana et al., 2006). The pathogenicity of the variant was confirmed by functional analyses and classified as a pathogenic variant according to the guideline of the American College of Medical Genetics and Genomics and the Association for Molecular Pathology (Richards et al., 2015). Neither pathogenic mutation nor reported polymorphism was found in genes causing NMS including *RYR1*, *DRD2/3*, *CYP2D6*, and *5HTR1A/2A* and causing hypersensitivity for PB including *CYP2C9/19*, *CYP3A5*, and *UGT1A4*. HLA genotypes associated with hypersensitivity for PB were not analyzed.

## 4 Discussion

The patient was diagnosed with *MAP2K1* variant-positive CFC at 45 years of age. Although she presented with psychomotor developmental delay and intellectual disability from infancy, she remained stable until the onset of epilepsy. The lack of typical congenital heart defects hindered the suspicion of CFC in this patient. However, seizures and musculoskeletal symptoms have been more commonly reported, and congenital heart defects are less frequent in *MAP2K1*-related CFC (Dentici et al., 2009; Leoni et al., 2021; Pierpont et al., 2022).

The first seizure in our patient occurred at 30 years of age. The phenotypes of epilepsy in *BRAF*-related CFC are classified into three groups according to the electroclinical features: early onset severe epileptic group causing developmental and epileptic encephalopathy, late-onset mild epileptic group primarily manifesting as controllable focal seizures, and seizure-free group above the age of 40 years (Battaglia et al., 2021). The present case exhibited late-age onset and uncontrollable seizures, which was different from the abovementioned three phenotypes. Among *MAPK1* variants, p.Y130 C/H/N is the most common and is associated with severe intractable epilepsy with multiple seizure types (Pierpont et al., 2022). Thus, although no seizures occur in childhood, the risk of adult-onset and refractory epilepsy might remain in *MAP2K1*-related CFC.

Little is known about the optimal antiseizure drugs for epilepsy in CFC. In the present case, polytherapy with VPA, LEV, and LCM was effective for combined generalized and focal epilepsy. In particular, the patient’s seizures responded well to LCM, which has a minimal risk of drug interactions, and is safe and effective. This finding is consistent with a recent publication showing relatively high efficacy scores of sodium-channel blockers in CFC (Kenney-Jung et al., 2024). Our patient experienced DIHS, an adverse drug reaction commonly associated with aromatic antiseizure drugs in Japanese patients, such as PB and carbamazepine (Hama et al., 2022). A combination of immunological reaction to a causative drug and human herpesvirus 6 reactivation results in DIHS. The prevalence of immunologically mediated adverse reaction is higher in Asian population than other areas, which associated with specific HLA alleles to each causative drugs. PB-induced adverse reaction is reported to be associated with HLA-B*51:01 in Japanese, and with HLA-A*01:01 or HLA-B*13:01 in Thai (Kaniwa et al., 2013; Manuyakorn et al., 2016; Rashid et al., 2022). Moreover, our patient had a history of NMS-like reactions caused by opioid and benzodiazepine. The clinical symptoms of NMS resemble to serotonin syndrome (SS) induced by serotonergic agents and malignant hyperthermia (MH) induced by anesthesia (Katus and Frucht, 2016). Those are differentiated by causative agents and by the presence of genetic factors such as mutations of *RYR1* in MH. Although triggered drugs are atypical in the present case, opioids can cause SS (Baldo, 2021) and benzodiazepines can cause NMS via cholinergic pathway (Al Rihani et al., 2021; Takahashi et al., 2022). There are supposed risk factors for these adverse events including dehydration, infection, and iron deficiency. In addition, the patients with intellectual disability, chromosomal abnormality, and neurogenerative diseases are reported to have susceptibility (Viejo et al., 2003). To the best of our knowledge, there is no known relationship between CFC and hypersensitivity to specific drugs.

Our patient appeared older and her motor and cognitive functions gradually deteriorated in her early 30 s. Obviously, the intractable epilepsy will adversely affect neuropsychiatric status. The osteoporotic thoracolumbar compression fractures led to a decrease in daily activity. The ileus exacerbated her nutritional status. These multiple factors can be associated with cognitive and motor decline. In addition, a case report of a patient with *BRAF*-related CFC delineated dementia-like symptoms and progressive decline from the early 30 s, despite well-controlled complications including neonatal-onset epilepsy (Cabrera et al., 2016). This case suggests frontal lobe dysfunction and the possibility of accelerated aging. Clinical features resembling premature aging are reported in patients with Costello syndrome (another RASopathy) and RAS-MAPK pathway can be associated with senescence (Engler et al., 2021). We should consider the possibility of worsening of psychomotor functions and complications in patients with CFC above the age of 30 years, irrespective of a stable state.

## 5 Patient perspective

We could observe long-term course of an adult patient with CFC caused by pathogenic *MAP2K1* variant, who manifested as adult-onset refractory epilepsy. Management of various complications has a significant influence on the prognosis of CFC. Seizure control is essential for better prognosis. Further information linked to genotypes is warranted to establish appropriate medication choices and minimize adverse drug reactions. This would aid in improving the outcomes in patients with CFC complicated by epilepsy.

## Data Availability

The original contributions presented in the study are included in the article/Supplementary Material, further inquiries can be directed to the corresponding author.
